# Analysis of treatment adherence and cost among patients with epilepsy: a four‐year retrospective cohort study in Pakistan

**DOI:** 10.1186/s12913-021-06085-0

**Published:** 2021-01-19

**Authors:** Muhammad Arif Asghar, Ahad Abdul Rehman, Muhammad Liaquat Raza, Yousra Shafiq, Muhammad Asif Asghar

**Affiliations:** 1 Department of Pharmaceutics, Institute of Pharmaceutical Sciences, Jinnah Sindh Medical University Karachi, Karachi, Pakistan; 2 Department of Pharmacology, Institute of Pharmaceutical Sciences, Jinnah Sindh Medical University Karachi, Karachi, Pakistan; 3 Department of Pharmacology, Faculty of Pharmacy, Shaheed Benazir Bhutto Dewan University, Karachi, Pakistan; 4 Institute of Neurophysiology, Charité – Universitätsmedizin Berlin, Berlin, Germany; 5 Food and Feed Safety Laboratory, Food and Marine Resources Research Centre, PCSIR Laboratories Complex, Shahrah-e-Salimuzzaman Siddiqui, Off University Road, 75280 Sindh, Pakistan

**Keywords:** Antiepileptic drugs, Adherence, risk factors, Cost analysis, Pakistan

## Abstract

**Background:**

The adherence pattern of antiepileptic drugs (AEDs) among patients with epilepsy is relatively lower in the United States and different European countries. However, adherence and cost analysis of AEDs in Asian countries have not been thoroughly studied. Therefore, the present study aimed to analyze the cost and adherence of AEDs and its associated factors in patients followed in Pakistan.

**Methods:**

Data from prescriptions collected from patients with epilepsy who have visited the Outpatient Department (OPD) of different tertiary care hospitals at the cosmopolitan city of Karachi, Pakistan from December 2015 to November 2019. The mean follow-up period for each participant was about 22 months. Pairwise comparisons from Cox regression/hazard ratios were used to assess the predictors of adherence. Direct costs of AEDs were calculated and presented as the annual cost of drugs.

**Results:**

A total of 11,490 patients were included in this study, 51.2 % were male and 48.8 % were female with a mean age of 45.2 ± 15.8 y. Levetiracetam was found as the most prescribing AED in all study participants (32.9 %). Of them, 49.1 % of patients continued their initial recommended treatment. However, 31.3 % of patients have discontinued the therapy, while, 19.6 % were switched to other AED. Adherence with initial treatment was more profound in male (57.4 %) patients, compared to female with a mean age of 44.2 years. Lamotrigine users (60.6 %) showed a higher tendency to retain on initially prescribed drugs. The total cost of epilepsy treatment in the entire study cohort was 153280.5 PKR ($941.9). By applying the Cox regression analysis, it can be observed that the patients with increasing age (OR, 2.04), migraine (OR, 2.21), psychiatric disorders (OR, 4.28), other comorbidities (OR, 1.52) and users of other than top five prescribing AEDs (2.35) were at higher risk of treatment discontinuation. However, levetiracetam (OR, 0.69), valproic acid (OR, 0.52), carbamazepine (OR, 0.81), lamotrigine (OR, 0.80) or lacosamide (OR, 0.65) users have more chances to continue their initial therapy.

**Conclusions:**

Similar to western countries, the majority of patients with epilepsy exhibited low adherence with AEDs. Various associated factors for improving adherence were identified in this study.

## Introduction

Epilepsy is a chronic brain disorder characterized by recurrent episodic seizures attacks and other psychiatric and somatic consequences [1]. Seizure’s development correlates with age because the process of aging itself can be a huge risk factor for seizures [2]. Therefore, progressive aging with time can be associated with an increase in the prevalence of epilepsy. The prevalence of this disease among the general population ranges from 2.3 to 15.9 per 1,000 in developed countries and from 3.6 to 15.4 per 1,000 in developing and under-developing countries [3]. According to the World Health Organization (WHO), around 50 million populations globally have been diagnosed with epilepsy [4]. One in 100 people currently lives with epilepsy in Pakistan as it belongs to the developing countries [5].

The sole choice of treatment available for the majority of epilepsy patients is antiepileptic drug (AED) therapy. Because epilepsy is characterized by sporadic symptoms (i.e., seizures) rather than a continually perceptible phenomenon like pain, the patient is required to take medications which do not have immediately obvious benefit. However, the consequences of nonadherence with treatment may not appear immediately. Thus, patients who miss doses or stop taking AED without experiencing adverse effects may erroneously conclude that medication adherence is not important [6].

Researches have shown that altering or missing AED dosages can have adverse reactions, like increase chance of seizure recurrence. Mattson et al. reported that more than one-quarter of seizures occurred at or before reports of inadequate intake of AEDs [7]. While Stanaway et al. found that more than one-third of epileptic seizures were due to either missed or inadequate administration of AEDs [8]. Furthermore, epilepsy patients must take daily AED for years or their lifetime.

The ability to follow initially recommended regimen for long periods is considered as treatment adherence. It may be affected by the variability in the frequency of seizures within a single patient’s course. Periods of exacerbation may induce fatalism and long periods of remission may induce complacency [6]. These factors complicate the assessment of treatment adherence and also the task of improving adherence. Failure to follow initially recommended AED regimen is known to be fairly widespread amongst patients with epilepsy. It has been reported that 25 to 75 % of patients with epilepsy, fail to adhere to initially prescribed AEDs [9–12]. Various study on analysis of medications usage produced significant impact on prescribing practices of medical practitioners [13, 14].

Previously, pharmacoeconomics, adherence and persistence with AEDs have been reported using various methods in different countries [15–19]. In our previous study, we have reported the prescribing patterns of AEDs in Southern Pakistan [13]. However, according to our literature study, in Pakistan, no such study was conducted regarding the patient’s adherence and cost analysis of AEDs. Therefore, this study aimed to assess the analysis of treatment adherence and cost analysis of AEDs in patients with epilepsy.

## Methods

### Study design

This cohort study was conducted from December 2015 to November 2019. Data were collected from patients with epilepsy who received AEDs for the first time. Patients who received following top five prescribed anti-epileptic drugs as descending order are as follows, levetiracetam, valproic acid, carbamazepine, lamotrigine, lacosamide while phenobarbital, phenytoin, gabapentin, pregabalin and oxcarbazepine were considered as others AEDs [13]. Data of participants who died or left from the study due to any reason were not included. Besides, patients with filled prescriptions for two or more AEDs used as polytherapy were also excluded.

### Data source

The ethical board of Ziauddin Hospital, Karachi, Pakistan has approved this cohort study with reference to our previous study. The prescriptions and other demographics were collected from patients with epilepsy who visited Neurology wards of different tertiary care hospitals of Sindh; the second largest province of Pakistan. It has numerous ethnic groups that represent the whole population of Pakistan. The sample size was calculated using statistical software Open Epi (Version 2.3.1), 85 % anticipated frequency at 95 % confidence interval (CI) with 5 % margin of errors [20]. Pharmacists collected the photocopies of prescriptions from patients with epilepsy after taking their consent at the time of medicine dispensing using a convenient sampling technique. Data including demographics, medical history, physical examination and any other comorbidity were abstracted from the patient’s previous medical profile without interviews. All cases of patients with epilepsy irrespective of age, gender and ethnicity were included on the following inclusion criteria; (i) confirmed the diagnosis of epilepsy (ii) At least one seizure episode was occurred during the previous 6 months with a currently used regimen of AEDs (iii) filled consent form.

People who used AEDs as polytherapy, or used herbal medications and pregnant women were placed in our excluding criteria. The following data was also written on each prescription for our record; hospital name, patient’s name, gender and age, confirmed diagnosis, medication regimen, treatment duration and next visit date for follow up.

### Analysis of treatment adherence

The mean follow-up period for each participant was 22.3 ± 4.2 months with a range of 16.5 to 36 months, while, 3.1 % was the drop rate during the study period. Treatment adherence is defined by the World Health Organization (WHO) as “the degree to which the patient corresponds with the agreed recommendations from a health care provider for a specified period” [21]. All studied participants were divided into three main categories: continuers, switchers and discontinuers. Patients who continued their initial drug treatment prescribed by practitioners in our defined follow up period called as continuers. Patients who switched to other AEDs from initially prescribed drugs considered as switchers. Lastly, who discontinued their initial therapy termed as discontinuers.

### Cost analysis

Direct costs of AEDs were calculated and presented as the annual cost of drugs for each patient individually as cost of continuer, switcher and discontinuer. The costs are expressed into the currency of Pakistani Rupees (PKR) and United States Dollars (USD) i.e. $1 = 162.70 Rupees (Rs.) on April 19th, 2020. The mean cost of studied AEDs, weighted for the dose per day prescribed by the practitioner for each epileptic patient, was Rs.40.3 for levetiracetam, Rs.10.2 for valproic acid, Rs.3.7 for carbamazepine, Rs.63.0 for lamotrigine, Rs.52.0 for lacosamide and Rs.21.0 for other above-defined five AEDs.

### Statistical analysis

All data obtained from patients of continuer, switcher and discontinuer are expressed as their mean values ± standard deviation. Statistical significant differences in all studied variables were analyzed by applying Chi-square statistical test using SPSS (version 23) software. Multiple risk factors or hazard ratios were calculated for the patient’s adherence to treatment using binary cox regression analysis (hazard ratio = 1). All p-values are two-sided tailed with *p* < 0.05 was considered as statistically significant.

## Results

A total of 13,565 “N” patients with epilepsy were enrolled for this study. After initial scrutiny, 2075 (15.2 %) participants were excluded because 1017 (7.5 %) were using those AEDs not included in this study, 624 (4.6 %) were taking drugs as polytherapy while 434 (3.1 %) left the study during the follow-up period. Thus 11,490 “N” patients were included in this study, of them, 5883 (51.2 %) and 5607 (48.8 %) were male & female respectively. The participant’s mean age was 45.2 ± 15.8 years (ranged 8–82 years). Table 1 showed that levetiracetam was the most commonly used AED (32.9 %), followed by valproic acid (21.1 %), carbamazepine (14.0 %), lamotrigine (11.2 %), lacosamide (9.3 %) and others AEDs as defined above (11.5 %). Patient demographics and characteristic data using different AEDs are also presented in Table 1.

**Table 1:** Demographics and characteristics data of epilepsy patients by different anti-epileptic drugs used as monotherapy

	Levetiracetam	Valproic acid	Carbamazepine	Lamotrigine	Lacosamide	Others
Patients* [N (%)]	3780 (32.9)	2424 (21.1)	1608 (14.0)	1288 (11.2)	1068 (9.3)	1322 (11.5)
Mean age** [y (SD**)]	55.2 (22.4)	10.4 (3.2)	21.5 (14.2)	45.1 (21.8)	13.9 (8.0)	52.4 (18.3)
Gender* (%) M/F	51.4/48.6	41.7/58.3	51.1/48.9	29.2/69.8	50.7/49.3	49.2/51.8
Patients with migraine** [N (%)]	645 (5.6)	234 (2.0)	848 (7.3)	26 (0.2)	102 (0.8)	472 (4.1)
Psychiatric disorders** [N (%)]	552 (4.8)	427 (3.7)	687 (5.9)	19 (0.1)	98 (0.8)	734 (6.3)
**Other comorbidities** [N (%)]	321 (2.7)	147 (1.2)	23 (0.2)	18 (0.1)	52 (0.4)	845 (7.3)

Phenobarbital, phenytoin, gabapentin, pregabalin and oxcarbazepine were categorized as others AEDs

Total (11,490 N) Patients

*SD* Standard deviation

*P < 0.05; **P < 0.005

In our study population, we found, that the highest percentage was of continuer’s i.e.49.1 %, followed by discontinuers (31.3 %), while the least percentage belonged to the switcher group (19.6 %). Demographics and adherence patterns of patients with epilepsy are presented in Table 2. It was observed that adherence of male participants with initial drug regimen was found much higher compared to female with the mean age of continuers participants was 44.2 years. It was also found that the mean duration of first-line treatment was around 165 days.

**Table 2:** Demographics and persistence patterns of epilepsy patients

	Continuers	Switchers	Discontinuers
Patients* [N (%)]	5641 (49.1)	2252 (19.6)	3597 (31.3)
Mean Age** [% (SD**)]	44.2 (17.3)	26.7 (7.8)	58.8 (24.2)
Gender* (%) M/F	57.4/42.6	58.2/41.8	42.8/57.2
Patients with migraine* [N (%)]	310 (2.7)	563 (4.9)	687 (5.9)
Psychiatric disorders** [N (%)]	137 (1.2)	689 (6.0)	999 (8.7)
Other comorbidities* [N (%)]	207 (1.8)	379 (3.3)	586 (5.1)

Total (11,490 N) Patients

*SD *Standard deviation

*P < 0.05; **P < 0.005

Table 3 presented the treatment adherence with the top five prescribing AEDs used as monotherapy. Adherence with initial treatment was observed much higher in patients using lamotrigine (60.6 %), lacosamide (57.5 %) and levetiracetam (54.1 %) compared to other AEDs users. However, discontinuation was found much higher in patients (56.2 %) who used drugs other than the top five prescribed AEDs. Valproic acid users were more substantial to switch their treatment to other drugs i.e. (33.5 %) in this study.

**Table 3:** Persistence patterns of top five prescribing anti-epileptic drugs as monotherapy

	Continuers N (%)	Switchers N (%)	Discontinuers N (%)
Levetiracetam**	2042 (54.1)	514 (13.5)	1224 (32.4)
Valproic acid**	1158 (47.8)	814 (33.5)	452 (18.7)
Carbamazepine*	745 (46.3)	299 (18.6)	564 (35.1)
Lamotrigine**	780 (60.6)	203 (15.7)	305 (23.7)
Lacosamide*	614 (57.5)	144 (13.5)	310 (29.0)
Others*	299 (22.6)	280 (21.2)	743 (56.2)
Total*	5638 (49.0)	2254 (19.7)	3598 (31.3)

Phenobarbital, phenytoin, gabapentin, pregabalin and oxcarbazepine were categorized as others AEDs

Total (11,490 N) Patients

*P < 0.05; **P < 0.005

The total cost of epilepsy treatment in the entire study cohort was Rs.153281.0 ($992.9), in which Rs.69423.0 ($426.6) belongs to continuers. Likewise, Rs.54490.9 ($334.9) cost by switchers, while discontinuers shared the lowest cost bracket of Rs.29366.6 ($180.4) with the percentage shares of 45.2 %, 35.5 % and 19.1 % respectively **(**Table 4**)**. The annual mean cost of levetiracetam treatment was Rs.9004.9 ($55.3), to Rs.4545.5 ($27.9) for valproic acid, to Rs.3224.2 ($19.8) for carbamazepine, to Rs.14075.5 ($86.5) for lamotrigine, to Rs.13010.6 ($79.9) for lacosamide and Rs.7232.8 ($44.4) for other drugs for epilepsy. For adherence, Rs.11570.5 ($71.1) was the annual average cost of continuers. However, Rs.9081.8 ($55.8) and Rs.4894.4 ($30.0) were accounted towards switchers and discontinuers respectively.

**Table 4:** Mean annual treatment cost of each epilepsy patient with respect to top five prescribing anti-epileptic drugs as monotherapy

	Mean cost for Continuers		Mean cost for Switchers		Mean cost for Discontinuers		Mean cost for study cohort	
	**PKR**^**a**^ **± SD**^**b**^	**USD**^**c**^ **± SD**	**PKR ± SD**	**USD ± SD**	**PKR ± SD**	**USD ± SD**	**PKR ± SD**	**USD ± SD**
**Levetiracetam**	14709.5 ± 3297.2	90.4 ± 20.2	6058.7 ± 1435.3	37.2 ± 8.8	6246.5 ± 1278.2	38.3 ± 7.8	9004.9 ± 2003.5	55.3 ± 12.3
**Valproic acid**	3723.1 ± 912.2	22.8 ± 5.6	8425.3 ± 2038.4	51.7 ± 12.5	1487.6 ± 437.6	9.14 ± 2.6	4545.5 ± 1129.4	27.9 ± 6.9
**Carbamazepine**	1350.5 ± 405.7	8.3 ± 2.4	7468.6 ± 1974.2	45.9 ± 12.1	853.7 ± 237.9	5.2 ± 1.4	3224.2 ± 872.6	19.8 ± 5.3
**Lamotrigine**	22995.2 ± 4397.3	141.3 ± 27.0	9574.5 ± 2044.9	58.8 ± 12.5	9657.2 ± 1949.2	59.3 ± 11.9	14075.5 ± 2797.1	86.5 ± 17.1
**Lacosamide**	18980.1 ± 3743.6	116.6 ± 23.0	11478.0 ± 2187.3	70.5 ± 13.4	8574.1 ± 1887.3	52.6 ± 11.5	13010.6 ± 2606.0	79.9 ± 16.0
**Others**	7665.0 ± 1825.5	47.1 ± 11.2	11485.8 ± 2295.6	70.6 ± 14.1	2547.6 ± 578.7	15.6 ± 3.5	7232.8 ± 1566.6	44.4 ± 9.6
**Total Mean Cost**	11570.5 ± 2430.2	71.1 ± 14.9	9081.8 ± 1995.9	55.8 ± 12.2	4894.4 ± 1061.4	30.0 ± 6.5	8515.5 ± 1829.2	52.3 ± 11.2
**Total annual cost**	**69423.0 ± 14581.5**	**426.6 ± 89.6**	**54490.9 ± 11975.7**	**334.9 ± 73.6**	**29366.6 ± 6368.9**	**180.4 ± 39.1**	**51093.5 ± 10975.3**	**314.0 ± 67.4**

Phenobarbital, phenytoin, gabapentin, pregabalin and oxcarbazepine were categorized as others AEDs

^a^Pakistani rupees

^b^Standard deviation

^c^United States Dollar

^*^1 USD = 154.38 PKR (At: 27-Jan-2020)

The inferential statistical analysis using the chi-square test showed the significant differences found in adherence of patients with epilepsy treatment with respect to all study variables and AEDs at *P* < 0.05 or *P* < 0.005. Moreover, the results of the Cox regression analysis indicated that patients with psychiatric disorders (OR, 4.28), migraine (OR, 2.21) other co-morbidities (OR, 1.52) and increasing age (OR, 2.04) were at higher risk of discontinuation from epilepsy treatment **(**Table 5**).** Users of levetiracetam (OR, 0.69), valproic acid (OR, 0.52), carbamazepine (OR, 0.81), lamotrigine (OR, 0.80) or lacosamide (OR, 0.65) have more chances to continue their treatment with the same drug. And users of other than these five AEDs have poor adherence with the odds ratio of 2.35.

**Table 5:** Statistical analysis of epilepsy patient’s adherence towards treatment using multiple linear cox regression analysis

Risk factors	Cox Regression Analysis		
	Control^**a**^/Cases^**b**^	OR^**c**^	CI^**d**^ (95%)
**Age**		2.04	(1.61-2.58)
**Patients with migraine**			
Yes	310/687	2.21	(1.79-2.74)
No	5331/2910	1.00	
**Psychiatric disorders**			
Yes	137/999	4.28	(3.63-4.93)
No	5504/2598	1.00	
**Patients with other comorbidities**			
Yes	207/586	1.52	(1.29-1.75)
No	5434/3011	1.00	
**Class of drugs**			
Levetiracetam	2042/1224	0.69	(0.55-0.86)
Valproic acid	1158/452	0.52	(0.46-0.58)
Carbamazepine	745/564	0.81	(0.69-0.96)
Lamotrigine	780/305	0.80	(0.68-0.95)
Lacosamide	614/310	0.65	(0.51-0.82)
Others	299/743	2.35	(1.93-2.88)

Phenobarbital, phenytoin, gabapentin, pregabalin and oxcarbazepine were categorized as others AEDs

^a^Continuers, ^b^Discontinuer, ^c^Odds Ratios, ^d^Confidence interval

## Discussion

In the present study, cost analysis and treatment adherence among patients with epilepsy were assessed. The use of levetiracetam was found much higher in comparison with other medications used to treat epilepsy which might be due to its efficacy and safety profile data in epileptic patients [22]. There was a significant difference in mean age among different AEDs users and frequent use of valproic acid was observed in young children. It could be due to a higher incidence of generalized epilepsy in early age. However, the use of valproic acid has been already prohibited due to its black box warning [23]. It is reported in previous studies that patients with epilepsy have more chances of increasing the prevalence of other comorbid conditions in comparison with the general population [24–26]. High incidence ratios of migraine, anxiety, depression, asthma, heart diseases, chronic bronchitis and diabetes were associated with patients having epilepsy [27]. In this study, carbamazepine and levetiracetam were the two most prescribing AEDs in migraine and other psychiatric patients. However, valproic acid is the only FDA approved AED for the prophylactic treatment of migraine [28]. In addition, the study also reported the use of lamotrigine and levetiracetam as prophylactic therapy in migraine [29]. Use of AEDs with psychiatric conditions becomes very challenging due to multiple reasons such as AEDs potentiate the symptoms of psychosis and also augmented the side effects of antipsychotics i.e. SSRIs induces hyponatremia when used with carbamazepine [30].

Unfortunately, not every patient with epilepsy achieves the desired therapeutic outcomes with the initial regimen. Treatment failure may result from inappropriate drug selection and doses, and poor adherence to the therapeutic regimen. Non-adherence to AEDs has been associated with a greater rate of mortality, increased hospital emergency visits, economic burden, reduced job-related efficiency and worsens the quality of life [31]. The risk of death was found to be three folds higher during quarters of nonadherence with AEDs [32]. Moreover, nonadherence to AED regimens makes epilepsy care much more expensive. Faught et al. also reported that additional cost per quarter of $4623 for each non-adherent patient compared with an adherent patient [32]. In this study, more than 50 % of the study population showed nonadherence with the initial regimen and also a negative association between discontinuation and patient age. To have a positive effect on the quality of life and to avoid side effects associated with AEDs, patients must adhere to initial prescribed AED on a long-term basis, usually for at least several years [6]. In agreement with present study findings, several studies have shown that adherence to AED is poor, varying between 30 % and 80 % [9, 33–35]. Furthermore, the mean duration of adherence with the initial regimen was around 165 days which is much lesser than the reported values of Divino et al. and Lai et al. i.e. 186 and 218 days in the USA and Taiwan respectively [16, 36].

In our study, the 31.3 % discontinuity with AEDs was observed, which is much greater in comparison with previously reported data that claims 29.18 % of patients showed complete discontinuation of AEDs. This study demonstrated that males more adhered to their treatment in comparison with females and these findings are paralleled with previous observation [37]. Women were found often more negative than men about the use of drugs which could be due to the reason that women are more sensitive to the ADRs [38].

Furthermore, discontinuation ratio was found much higher in patients receiving first-generation AEDs. It is in comparison to those receiving five top most new AEDs which corresponded with the reported findings of Jacob et al. in 2017 [39]. The potential reason for such results could be fewer drug interactions and side effects of new AED than old AED [39]. The higher switching ratio with valproic acid may be elucidated by the fact that it should be avoided or replaced in female of childbearing age due to its teratogenicity [40]. Several factors may contribute to such nonadherence behavior associated with reported AEDs including age, gender, comorbidities and use of concomitant medications [41]. In line with previous data, gender-related influence on the use of different AEDs was also observed in the present study. It has been observed that the use of lamotrigine in female was found more frequently than male, which could be due to its negligible teratogenic effects [42]. Few observations of the present study were supported by SANAD trials, as lamotrigine users showed better adherence in comparison with carbamazepine [43].

Evaluation of cost-effectiveness of treatment was done by estimating mean therapy. Treatment adherence, AED and patient’s age were those factors that greatly affect the overall cost of epilepsy treatment. The switching pattern and discontinuity of AEDs have also been associated with the economic burden. Therefore, following previous studies, we also found that the overall mean cost of continuers was much higher when compared with discontinuers and switchers [15, 44, 45]. Significant differences were observed in the total cost of treatment among continuers, switchers and discontinuers concerning each AED. These differences in cost were due to the high ratio of switching of drugs from one AED to another. Treatment adherence should be improved by choosing the more appropriate drug resources, to avoid the long term complications and conversion of patients in a chronic state [46]. Assessing the mean cost of epilepsy therapy is a key feature to evaluate the cost-effectiveness of alternative pharmacologic agents in epilepsy treatment. The overall cost of epilepsy treatment was greatly affected by the type of AED, age, persistence and adherence pattern. The mean cost of epilepsy treatment was high in the case of lamotrigine and lacosamide in comparison to other AEDs. Medications used in epilepsy treatment are generally expensive, especially newer AEDs being the most expensive class of AEDs in the pharmacotherapy of epilepsy [47]. However, low cost does not mean that the drug has to be the first choice in all subjects because the patient’s preference also depends on drug efficacy, safety and percentage tolerability. Hence, patients showed greater continuity with new AEDs due to their efficacy and high tolerability. On the other hand, it has been observed that the cost of treatment was immensely reduced in those who switched from new AEDs to older ones. It has been reported that such nonadherence with new AEDs and switching to other has been massively associated with increased cost in primary care [48]. Similarly in German study, Willems et al., in 2019 reported that the mean price of first-generation AEDs increased up to 133 % between 2000 and 2017 [49]. In addition, Willems et al., in 2018 also stated that the early retirement was the main cost factor despite improved labor market opportunities [50].

Statistical analysis using the Chi-square test at *P* < 0.05 showed that all studied variables caused significant effects on treatment adherence. Odds ratio (OR) with 95 % CI using Cox regression analysis was applied to estimate the treatment adherence with studied variables. Higher the value of ORs indicates a greater probability of treatment nonadherence and discontinuation. The statistical analysis showed that the increasing age, migraine, psychiatric disorders and other comorbidities subjects were at higher risks of treatment nonadherence and discontinuation. Moreover, the possibility of discontinuation was found much higher in patients who used AEDs other than the top five prescribing newer AEDs. However, higher chances of treatment adherence were observed in new AEDs users with low ORs values.

Final considerations are much essential part of this discussion concerning the limits of this study. The polling of results across the study design may not fully appropriate, which is an important point of this study such as geographic and regional population, epilepsy duration, the success of treatment, dosage of drugs and many others. Due to this limitation, we refrained from combining the result of observational studies. Moreover, fluctuation in the severity of epilepsy was found in many patients that may cause termination of prophylaxis medication. On the point of fact, the study data is not fully warranted for treatment adherence and discontinuation pattern. Despite all these limitations, we believe that our study findings provide a broad representation of treatment adherence and cost analysis of different AEDs. Further, it highlights the need for further studies to improve the patient’s adherence to epilepsy therapy.

## Conclusions

Overall, the share of adherence with AED in patients with epilepsy is lower than 50 % after four years of treatment. Age, gender, co-morbidities and drug characteristics are associated with this adherence. The mean cost of AEDs increases due to switching from one AED to others. New options with cost-effective, improved tolerability and drugs with fewer dosing intervals may improve patient adherence towards therapy. Adherence with treatment should be considered as an endpoint in future observational studies exploring the more concise pattern and usage of such therapies. In all chronic disorders, treatment adherence remains a big challenge and such conditions may require the use of drugs for the whole or major part of life.

## Data Availability

The data sets used and/or analyzed during the current study available from the corresponding author on reasonable request.

## Abbreviations

AEDs: Antiepileptic drugs
CI: Confidence interval
OPD: Outpatient Department
**OR**: Odd ratio
PKR: Pakistani Rupees
**SPSS**: Statistical Package for the Social Sciences
SSRIs: Selective serotonin reuptake inhibitors
**USD**: United States Dollars
**WHO**: World health organization
